# Cadmium Toxicity Induced Alterations in the Root Proteome of Green Gram in Contrasting Response towards Iron Supplement

**DOI:** 10.3390/ijms15046343

**Published:** 2014-04-15

**Authors:** Sowbiya Muneer, Khalid Rehman Hakeem, Rozi Mohamed, Jeong Hyun Lee

**Affiliations:** 1 Department of Horticulture, College of Agricultural Life Sciences, Chonnam National University, 300 Young Bong-Dong Buk-Gu, Gwangju 500-757, Korea; E-Mails: sobiyakhan126@gmail.com (S.M.); leetag@chonnam.ac.kr (J.H.L.); 2 Faculty of Forestry, Universiti Putra Malaysia, Serdang 43400, Selangor, Malaysia; E-Mail: rozimohd@upm.edu.my

**Keywords:** cadmium toxicity, iron suppliment, proteomics

## Abstract

Cadmium signifies a severe threat to crop productivity and green gram is a notably iron sensitive plant which shows considerable variation towards cadmium stress. A gel-based proteomics analysis was performed with the roots of green gram exposed to iron and cadmium combined treatments. The resulting data show that twenty three proteins were down-regulated in iron-deprived roots either in the absence (−Fe/−Cd) or presence (−Fe/+Cd) of cadmium. These down-regulated proteins were however well expressed in roots under iron sufficient conditions, even in the presence of cadmium (+Fe/+Cd). The functional classification of these proteins determined that 21% of the proteins are associated with nutrient metabolism. The other proteins in higher quantities are involved in either transcription or translation regulation, and the rest are involved in biosynthesis metabolism, antioxidant pathways, molecular chaperones and stress response. On the other hand, several protein spots were also absent in roots in response to iron deprivation either in absence (−Fe/−Cd) or presence (−Fe/+Cd) of cadmium but were well expressed in the presence of iron (+Fe/+Cd). Results suggest that green gram plants exposed to cadmium stress are able to change the nutrient metabolic balance in roots, but in the mean time regulate cadmium toxicity through iron supplements.

## 1. Introduction

Fe is abundant in the earth’s crust but exists as insoluble Fe (III) precipitates and is largely unavailable to plants, especially those which are growing in alkaline and calcareous soils. Fe uptake and transport in plants use two Strategies: Strategy I and Strategy II. Higher plants such as graminaceous monocots utilize the Strategy II mechanism and use iron acquisition in the form of phytosiderophores (PS), which solubilizes ferric Fe in the rhizosphere [1,2]. However, dicots utilize the Strategy I mechanism for Fe-uptake and transport. Leguminous plants are sensitive to Fe due to a symbiotic relationship thus, often face Fe-deficiency. Due to Fe-deficiency, legumes are primarily affected for their seed development [3] which are rich in starch and protein content. Fe-deficiency in plants lead to several other physiological alterations like photosynthesis, respiration, nitrogen fixation, DNA synthesis and chlorophyll formation [4–6].

Cadmium (Cd) is one of the most toxic metals with no biological function. It is readily taken up by roots, probably in competition with other divalent ions, and restricts plant growth and development [7,8]. As plants respond to Cd, alterations in protein expression patterns take place. Changes in the abundance of proteins could suggest a possible role under Cd stress [9,10]. Cadmium is well known for its phytotoxicity, which is associated with number of morphological, physiological and biochemical events [11]. Cadmium exerts its toxic effects through its high affinity for sulfhydryl groups in proteins and other biological molecules and induces oxidative stress by inhibiting reactive oxygen species (ROS) detoxifying enzymes [12]. These responses are direct consequences of changes in gene expression. Cadmium also induce a series of alterations that can influence the proteome of roots since roots are the first organ in plants to experience toxicity [13,14]. Monitoring protein expression changes under Cd stress is one way of identifying the genetic and molecular componets underlying Cd tolerant phenotypes.

Most of the important studies concering Cd-Fe interactions have been performed using Strategy I plants [15–19] but little is known about the response to cadmium toxicity in iron-deficient Strategy II plants, and few studies have been reported in barley [11] and rice [2]. Whereas interaction between Fe and Cd on leguminous plants are still lacking. Previously we identified proteome of leaf/root-nodule in green gram at lower concentration of CdCl_2_ (50 μM). Thus, the present study was to characterize the root proteome of green gram to higher concentration of CdCl_2_ as roots are the main part in plants which is primarily affected by metal toxicity. The classification of the proteins expressed by iron and cadmium combined treatment merits a good methodology to comprehend the possible roles of iron-nutrition in improving cadmium toxicity tolerance in leguminous plants. In this study, a gel based proteomics approach and a functional classification of the identified proteins was performed for the roots of *Vigna radiata* after 10 d of iron and cadmium combined treatments.

## 2. Results

### 2.1. Differentially Expressed Proteins in Roots by 2DE

**Figure 1.**
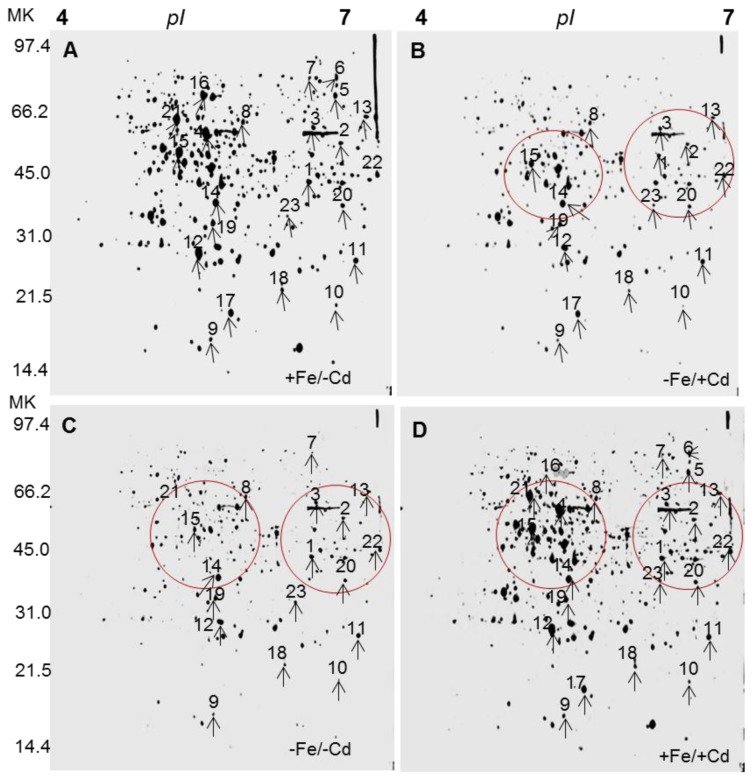
2D reference images of four Fe/Cd combined treatments: (**A**) Sufficient in iron and no cadmium (+Fe/−Cd, control); (**B**) Deprived iron and cadmium toxicity (−Fe/+Cd); (**C**) Deprived iron with no cadmium (+Fe/−Cd); and (**D**) Sufficient iron but cadmium toxicity (+Fe/+Cd) in the roots of *Vigna radiata* L. Proteins were separated in the first dimension on a nonlinear IPG strip, pH 4–7 and in 2nd dimension on 12% polyacrylamide SDS-gel. Quantitative image represents differentially expressed proteins. Red encircles indicate absence of protein spots with respect to that of control. Black arrows indicate comparison of protein spots to each other identified by MALDI-TOF.

After separation of proteins on 2DE, the protein spots obtained from Fe and Cd combined treatments were evaluated by comparing that of control (+Fe/−Cd) with the PD-Quest software with three replications. Five hundred proteins were reproducibly detected on 2D gels by Fe and Cd combined treatments. Twenty three protein spots (spot 1–23) were down-regulated under Fe-deprived condition regardless of Cd absence (−Fe/−Cd) or presence (−Fe/+Cd) (Figure 1B,C indicated by numbers). However, these down-regulated proteins were well expressed (similar to control level) in the presence of Fe (+Fe/+Cd). In addition, several proteins were absent (red encircled areas in 2D gels) under Fe-deprived conditions in the absence (−Fe/−Cd) and presence of Cd (−Fe/+Cd), but they were present to control level in the presence of Fe (+Fe/+Cd) compared to controls. Analogusly few other proteins were found very faint (down-regulated) (blue encircled areas in 2DE gels, not identified by MS) under Fe-deprived conditions in the absence (−Fe/−Cd) and presence of Cd (−Fe/+Cd), but they were present to control level in the presence of Fe (+Fe/+Cd) compared to controls.

### 2.2. Identification of Differentially Expressed Proteins in Roots

**Figure 2.**
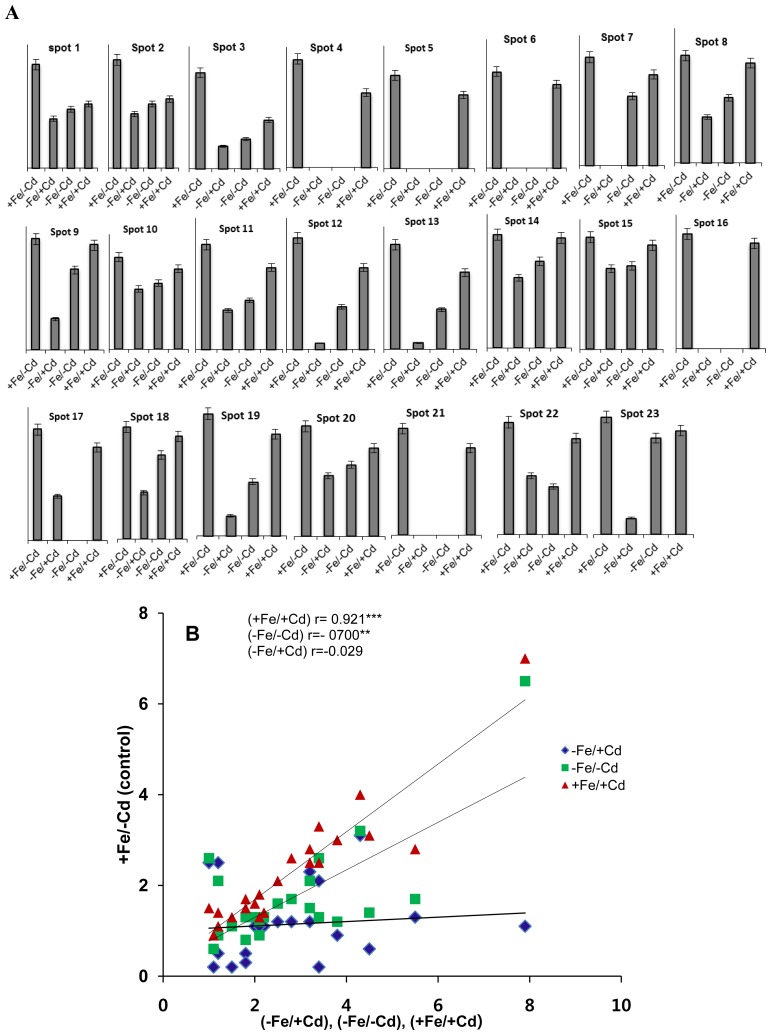
(**A**) Relative intensities expressed as fold change; (**B**) Linear correlation compared to control 10 days of Fe/Cd combined treatments, sufficient in iron and no cadmium (+Fe/−Cd, control), deprived iron and cadmium toxicity (−Fe/+Cd), deprived iron with no cadmium (+Fe/−Cd), and sufficient iron but cadmium toxicity (+Fe/+Cd) in the roots of *Vigna radiata* L. The relative intensity was quantified by PD-Quest software.

**Figure 3.**
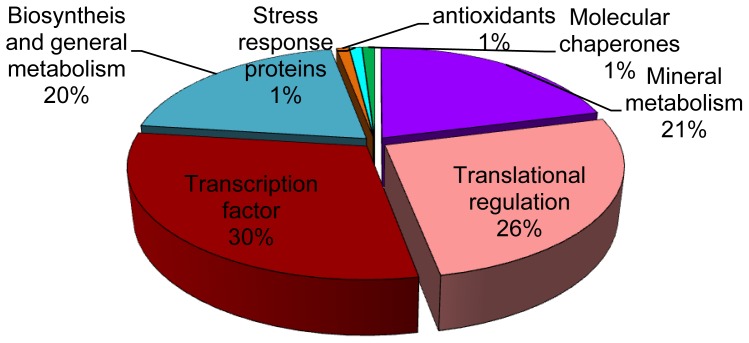
Functional classification of identified proteins from leaves analyzed by MALDI-TOF-MS as described by Beven *et al.* [20].

Twenty three proteins down-regulated under Fe-deprived in the absence of Cd (−S/−Fe) and in the presence of Cd (−Fe/+Cd) along with presence of Fe under Cd toxicity (+Fe/+Cd) were identified by MALDI-TOF-MS (Table 1). The five proteins related to mineral metabolism (Figure 3) down-regulated by 1–4-fold (Figure 2A) in absence (−Fe/−Cd) and presence of cadmium (+Fe/+Cd) identified were cysteine protease inhibitor (spot 5, 6), ethylene receptor (spot 7), magnesium chelate (spot 14), and calcium binding protein (spot 15). However, these proteins were well expressed and were increased by 1-fold (Figure 2) in the presence of iron (+Fe/+Cd) under cadmium toxicity condition.

Besides mineral metabolism related proteins, 13 other down-regulated proteins in absence (−Fe/−Cd) and presence of Cd (+Fe/+Cd) were related to transcriptional/traslational regulation or biosynthesis/general metabolsim and were identified as beta-ketoacyl-acyl carrier protein synthase III (spot 1), *C*-terminal processing protease (spot 2), DVL family (spot 3), polyglactouranase (spot 4), (*S*)-2 hydroxyacid oxide (spot 8), Putative cysteinyl-tRNA synthetase (spot 10) Small nuclear ribonucleoprotein f, putative (spot 11), Nucleotide-binding site leucine-rich repeat protein (spot 12), Retrotransposon protein (spot 13), Ribosomal protein L23 (spot 20), HD domain class transcription factor (spot 21), *C*-terminal processing protease, putative (spot 22) and (+) neomenthol dehydrogenase (spot 23). Whereas they were well expressed in iron nutrient plants in the presenec of cadmium (+Fe/+Cd).

Four other proteins related to antioxidants, stress response and molecular chaperons down regulated from Fe/Cd treated roots (−Fe/−Cd or/and −Fe/+Cd) were identified as heat shock protein (spot 16), Pathogenesis related protein (spots 17, 18) and glutathione S transferase omega like protein (spot 19) were, however, up-regulated to control level in presence of iron (+Fe/+Cd).

The over all threshhold of all differentially expressed and identified proteins were also correlated with each other by co-relation cofficients (Figure 2B) by comparing protein spots from control roots (+Fe/−Cd) with protein spots from roots under Fe-deprivation with (−Fe/+Cd) or without (−Fe/−Cd) along with protein spots from roots under Cd-toxicity with presence of Fe (+Fe/+Cd). Control roots (+Fe/−Cd) were closely related (*p* < 0.001) with roots under Cd-toxicity in presence of Fe (+Fe/+Cd) and (*p* < 0.01) with protein spots under Fe-deprivation without (−Fe/−Cd) Cd and (*p* < 0.005) with Cd.

**Figure 4.**
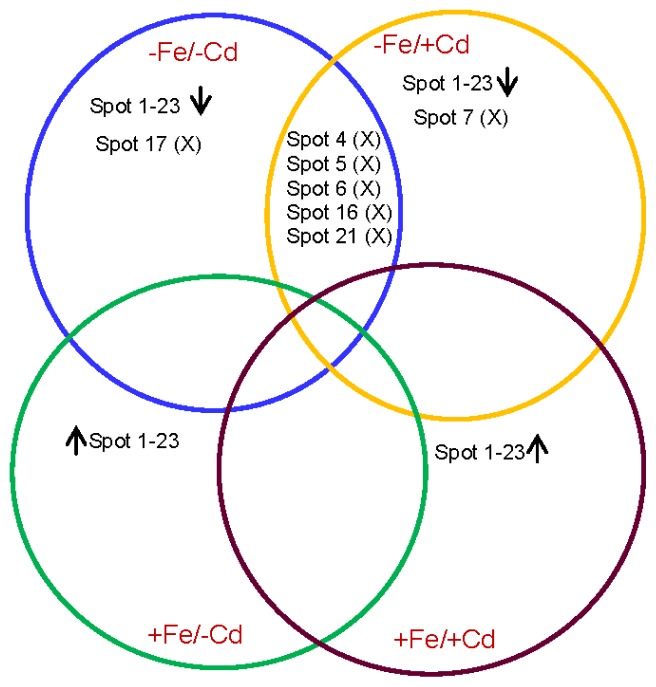
*Venn diagram* showing differential expression of root proteins of green gram under four combined Fe and Cd treatemnet. Numbers correspond to the protein spots present in 2DE patterns of +Fe/+Cd, −Fe/−Cd, −Fe/−Cd and +Fe/+Cd. The *overlapped* portions with “X” denote commonly absent proteins. *Upward* and *downword* arrows denote increased or decreased protein expression under four treatments.

All identified proteins were classified by Beven *et al.* [20], and were grouped into seven classes (Figure 4) as 30% transcription factor, 26% translational regulation, 21% mineral metabolism, 21% biosynthesis and general metabolism, 1% antioxidants, stress responses and molecular chaperones.

### 2.3. Comparison of Diffrentailly Expressed Proteins among Four Treatments

Out of 23 diffentially expressed protein spots, five proteins were commonly observed in roots under Fe-deprivation with (−Fe/+Cd) or without cadmium (−Fe/−Cd) (Figure 4). Under Fe-deficiency with or without cadmium, all these five proteins (spots 4, 5, 6, 16 and 21) were absent in both treatments however, were commonly present between control (+Fe/−Cd) and cadmium stressed root in presence of cadmium (+Fe/+Cd).

Similarly, proteins spots between 1 and 23 (except 4, 5, 6, 16 and 21) were down-regulated in −Fe/−Cd and −Fe/+Cd roots whereas they were up-regulated in +Fe/+Cd plants compared to control (+Fe/−Cd).

## 3. Discussion

The toxic effects of cadmium in plants can be recognized by the induction of oxidative stress leading to enhanced production of reactive oxygen species (ROS) and toxic substances. Exposure to cadmium elicited an oxidative burst in green gram roots indicated by the production of ROS (data not shown). ROS have been characterized as key factors in the response of plants to both biotic and abiotic stresses [21]. Our study reflects that iron deficiency as well as cadmium stress impairs the ability of plants to counterattack ROS. Further, the presence of cadmium under iron deficiency enhanced the intensity of oxidative stress whereas, were restored by the presence of iron even in the presence of cadmium toxicity. This indicates that iron might have helped the roots in controlling the formation of oxyradicals under both stresses because iron is important for expression of proteins which leads to conversion of singlet oxygen into hydrogen peroxide and finally into the water.

Proteomic analysis showed that iron and cadmium reduced protein spots in number (Figure 1). The loss of protein spots was observed under cadmium toxicity, mostly in the absence of iron (Figure 1B) and less in the presence of iron (Figure 1C), while it was largely restored in the presence of iron even under cadmium toxic conditions (Figure 1D). The loss of protein spots might be due to progressive depletion of biochemical pathways associated with signal transduction and gene regulation, in particular those involved in protein synthesis [22,23], and might be associated with an excessive production of ROS which leads to incorrect folding or assembly of proteins, and consequent protein degradation [24]. Recently, we estimated that iron resulted in strong induction of proteins by 40%–50% in mature leaves of oil seed rape [25]. This suggests that iron-nutrition retains the loss of proteins in roots by activating the induction of iron transporter mechanism [2] and by increasing *de novo* synthesis of proteins in cadmium toxic roots. In the iron-deprived roots (−Fe/Cd and −Fe/+Cd), twenty three proteins were down-regulated, but these down-regulated proteins were well expressed in the presence of iron (+Fe/+Cd) (Figure 1D). The loss of protein (down-regulation) in iron and/or cadmium toxic roots may reflect an overall reduction of biosynthesis of nutrient related proteins and the proteins which are related to transcription and translation. In our results 26%–30% of differently expressed proteins by iron and cadmium combined treatment was identified to be related to transcription and translational regulation such as beta-ketoacyl-acyl carrier protein synthase III (spot 1), *C*-terminal processing protease (spot 2), DVL family (spot 3), polyglactouranase (spot 4), (*S*)-2 hydroxyacid oxide (spot 8), Putative cysteinyl-tRNA synthetase (spot 10) Small nuclear ribonucleoprotein f, putative (spot 11), Nucleotide-binding site leucine-rich repeat protein (spot 12), Retrotransposon protein (spot 13), Ribosomal protein L23 (spot 20), HD domain class transcription factor (spot 21), *C*-terminal processing protease, putative (spot 22) and (+) neomenthol dehydrogenase (spot 23) (Table 1). These proteins were 1–5-fold downregulated in iron-deprived roots (more severely in the presence of cadmium), whereas they were expressed at the control level in the iron supplied roots (Figure 1). This may be caused by a stronger decline in iron transporters in the roots rather than that in Fe content. These results suggest that the consequent availability of cadmium may limit the activity of iron chelates and phytochelatins which are involved in the transport of iron [16,19] especially in green gram which is a high iron-demanding plant [5,6]. It can be thus concluded that down-regulation of transcriptional and translational proteins along with nutrient metabolism related proteins in cadmium toxic roots in the absence of iron is probably due to proteolytic loss of iron homeostatis [26–28] and the loss of enzymes which are involved in the formation of root nodules in leguminous plants such as leghemoglobin and ferritin [5]. In addition, some proteins related to stress response, molecular chaperons and antioxidants were downregulated by iron and cadmium combined treatment (Table 1) such as heat shock protein (spot 16), Pathogenesis related protein (spots 17, 18) and glutathione S transferase omega like protein (spot 19) but were induced to the control level in the presence of iron. This implies that after a prolonged period of cadmium toxicity, proteins which are particularly induced in stressed plants to mitigate oxidative stress did not chelate the toxicity of cadmium unless the iron supplement was given to the plants. These results indicate that iron supply under cadmium toxicity has a role in regulating the biosynthesis of stress responsive related proteins which are the main component involved in chelation of toxic metals and regulation of transcriptional/translational genes which are involved in the uptake and transport of iron from the soil via roots to other parts of plants [29–31] in green gram plants. Although the comparison between protein spots were made based on 2DE reference maps and identified proteins, we also made a clear comparison of protein spots by relatively overlapping protein spots among all the four treatments by venn diagram (Figure 3) to represent a reference maps between differentially expressed proteins. Interestingly, a great level of divergence in protein spots were observed in all the four treatments. This divergence might have arisen due to amino acid compositions and protein length. Our comparative analysis made a clear cut conclusion that iron supplementation is a positive influence on cadmium toxicity by up-regulating protein expression probably due to reducing the risks of oxidative damage at cellular level. Taken together, the results indicate that iron has a significant role in the proteome of roots by mitigating cadmium toxicity in green gram plants.

## 4. Methods

### 4.1. Plant Culture and Treatment

Surface-sterilized seeds of *Vigna radiata* L (Var. Sun-Hwa-Nok-Du, Seoul, Korea) were germinated on wet filter paper in Petri dishes at 30 °C in the dark for three days. After germination, plants were divided into four treatment groups consisting of: [+Fe/−Cd, control] sufficient Fe, no Cd-toxicity, [+Fe/+Cd] sufficient Fe, with Cd toxicity (300 μM), [−Fe/−Cd] Fe deprived, no Cd toxicity, and [−Fe/+Cd] Fe deprived Fe, with Cd toxicity (300 μM), with four replicates of each. High concentrations of CdCl_2_ (300 μM) were used in this study (in contrast to our previous studies in which plants were treated at 50 μM CdCl_2_ concentrations. A highly tolerant to metal toxicity plant species was used in this study and till now it is has not been reported whether highly concentrated Cd-toxic plants are able to mitigate the toxicity. In this experimental study, plants were grown in a 3 L plastic pot with hydroponic nutrient solution containing (at mM concentrations for the macro elements): 1.0 NH_4_NO_3_; 0.4 KH_2_PO_4_; 3.0 CaCl_2_; 1.5 MgSO_4_; 0.15 K_2_HPO_4_; 0.2 Fe-Na EDTA; and (μM for the micro elements): 14 H_3_BO_3_; 5.0 MnSO_4_·H_2_O, 3.0 ZnSO_4_·7H_2_O; 0.7 CuSO_4_·5H_2_O; 0.7 (NH_4_)_6_MO_7_O_24_; 0.1 COCl_2_. The nutrient solution was continuously aerated and renewed every three days. For Fe-deficient conditions the Fe was completely removed from the hydroponic solution by omitting Fe-Na EDTA. Natural light was supplemented with controlled lighting set at 200 μmol·m^−2^·s^−1^ at canopy height for 16 h·day^−1^. After 10 days treatment periods, roots were excised from the main plant and immediately frozen in liquid N_2_ before storage in a deep-freezer for further analysis.

### 4.2. Protein Extraction, 2-D SDS-PAGE and Image Analysis

Briefly, 1 g of roots tissues were grounded with pestle and mortar in liquid nitrogen according to method given by Qureshi *et al.* (2010) [32], homogenized with extraction buffer (pH 7.6) containing 40 mM Tris, 0.07% βME (beta mercapto ethanol), 2% PVP (Polyvinylpyrrolidone) and 1% Triton X100 at 4 °C by centrifugation at 15,000 rpm for 15 min. Following the centrifugation proteins were precipitated with 10% TCA/acetone overnight at −20 °C. The subsequent precipitate was washed with 80% chilled acetone containing 2 mM EDTA and 0.07% βME. The resultant protein pellet was solubilized in solubilisation buffer containing 9 M urea, 2 M thiourea, 4% CHAPS, 2% Triton X100, 50 mM DTT and 0.2% ampholine (pH 4–7) and a trace of bromophenol blue and were quantified by BSA. Three hundred microgram of each dissolved protein were rehydrate on 11 cm ready IPG strip (pH 4–7) (Bio-Rad, Hercules, CA, USA), followed by focussing for 72,000 volt hour using IPGphor isoelectric focussing (IEF), the IPG strips were equilibrated for 30 min in reduction buffer containg 50 mM Tris-HCl (pH 8.8), 6 M Urea, 30% (*v*/*v*) glycerol, 2% SDS and 10 mg/mL DTT, followed by alkylation buffer containing 25 mg/L idoacetamide. The 2nd dimensional SDS-PAGE was carried out using 12.5% polyacrylamide gels, stained with Coomassie Brilliant Blue (CBB-R250). Images were acquired at high resolution scanner (Gel doc; Bio-Rad) and analysed using PDQuest Software (V 8.0.1; Bio-Rad). After automated spot detection manual supervisison was carried out; followed by Tryptic digestion, MALDI-TOF MS and extensive data base serach.

The proteins of interest were excised manually from the gels, and washed with distilled water three times by a centrifugation for 1100 rpm at 22–24 °C for 10 min. The protein spots were treated with 50% acetonitrile after washing and centrifuged for 10 min at 22–24 °C at 1100 rpm. The protein spots were then treated with 1 M DTT and 50 mM ammonium carbonate, and incubated for 45 min at 38 °C. After incubation protein spots were treated with IAA and 55 mM ammonium carbonate, centrifuged again for 30 min at 22–24 °C at 1100 rpm and were vacuum dried for 15 min. The protein spot was then treated with trypsin and were kept at 37 °C overnight, After overnight digestion the petides were dried. Samples were analyzed using Voyager-DE STR MALDI-TOF mass spectrometer (PerSeptive Biosystems, Framingham, MA, USA). Parent ion masses were measured in the reflectron/delayed extraction mode. Peptide mass fingerprinting (PMFs) obtained from digested proteins were compared with PMFs in matrix science using MASCOT software (version 2.3; Matrix Science, London, UK), search was performed with all *viridiplante*.

## 5. Conclusions

From the above studies we concluded that iron supplement to plants exposed to cadmium toxicity, a novel strategy of proteomic analysis assisted by bioinformatics have a certain potential for identification of stress responsive proteins and the development of plants tolerant to abiotic stress, pathogens and disease. Identification of proteins and their encoding genes in legumes will definitely provide important information for the development of plants with better potential stress tolerance and greater N_2_-fixing efficiency.

## Figures and Tables

**Table 1.** Identification of differentially expressed proteins under Fe/Cd combined treatment in roots of *Vigna radiata* identified by MALDI-TOF MS.

Spot	Acession number	Homology	% coverage	Matched peptides	Mascot score	Mr value	Ther.*pI*/Exp.*pI*	Species
1	gi/35172183	beta-ketoacyl-acyl carrier protein synthase III	20	82	53	41,844	6.6/6.7	*Glycine max*
2	gi/89257673	*C*-terminal processing protease, putative	14	70	39	54,888	8.9/7.0	*Brassica olereaca*
3	gi/42571085	DVL family protein	25	28	38	6234	8.9/7.0	*Arabidopsis thaliana*
4	gi/255548021	Polygalacturonase, putative	25	61	38	26,835	5.4/5.5	*Ricinus communis*
5	gi/187473813	Cysteine proteinase inhibitor	72	44	39	6888	9.3/6.9	*Sonneratia griffithii*
6	gi/187473851	Cysteine proteinase inhibitor	72	44	39	6830	9.5/7.0	*Sonneratia griffithii*
7	gi/11935116	Ethylene receptor	57	63	60	12,773	7.5/7.0	*Populus trichocarpa*
8	gi/145333373	(*S*)-2-hydroxy-acid oxidase	18	60	38	34,382	7.7/7.0	*Arabidopsis thaliana*
9	gi/224112805	AP2/ERF domain-containing transcription factor	21	50	39	26,024	5.0/4.5	*Populus trichocarpa*
10	gi/110740110	Putative cysteinyl-tRNA synthetase	32	46	38	15,724	7.8/6.3	*Arabidopsis thaliana*
11	gi/255587821	Small nuclear ribonucleoprotein f, putative	30	28	49	9802	9.0/4.5	*Ricinus communis*
12	gi/379068466	Nucleotide-binding site leucine-rich repeat protein	27	73	68	30,937	5.0/5.0	*Rhododendron formosanum*
13	gi/77553861	Retrotransposon protein	42	64	40	16,417	8.7/6.8	*Oryza sativa*
14	gi/224178035	Magnesium chelatase	23	79	40	37,402	4.0/4.7	*Pyramimonas parkeae*
15	gi/15239904	Calcium binding protein	38	61	39	17,200	4.2/6.0	*Arabidopsis thaliana*
16	gi/55742654	Heat shock protein 70	20	44	80	75,480	5.1/5.0	*Galus gallus*
17	gi/34395061	Pathogenesis related protein1	39	61	88	16,417	4.6/4.5	*Oryza sativa*
18	gi/105990543	Pathogenesis related protein 2	30	48	55	16,518	4.8/4.5	*Zea mays*
19	gi/124301261	Glutathione S transferase omega like protein	32	106	42	37,448	5.7/5.0	*Medicago trancatula*
20	gi/125987561	Ribosomal protein L23	54	52	39	11,192	10/6.5	*Sorghum bicolor*
21	gi/302398837	HD domain class transcription factor	14	80	40	646,677	6/4.6	*Malus domestica*
22	gi/89257673	*C*-terminal processing protease, putative	14	88	39	54,888	8.9/7.0	*Brassica olerecea*
23	gi/357460053	(+) neomenthol dehydrogenase	23	70	66	32,472	6.3/4.2	*Medicago trancatula*

## Abbreviations

2DE: two dimensional gel electrophoresis
SDS-PAGE: sodium dodecyl sulfate poly acrylamide gel electrophoresis)
MALDI-TOF: matrix assisted laser desorption/ionization-time of flight
IPG: immobilized pH gradient
IAA: Iodoacetamide
DTT: dithiothriotol
